# Athermal strength of pure aluminum is significantly decreased by severe plastic deformation and it is markedly augmented by subsequent annealing

**DOI:** 10.1038/s41598-020-70160-5

**Published:** 2020-08-24

**Authors:** Takayuki Koizumi, Anna Kurumatani, Mitsutoshi Kuroda

**Affiliations:** 1 Faculty of Human Resources Development, Polytechnic University of Japan, Tokyo, 187-0035 Japan; 2 Graduate School of Science and Engineering, Mechanical Engineering, Yamagata University, Yonezawa, Yamagata 992-8510 Japan

**Keywords:** Mechanical properties, Metals and alloys

## Abstract

Over the last two decades, it has been considered that fine crystal grains produced by severe plastic deformation (SPD) lead to an extraordinarily high metal strength. The present study reveals that this understanding is basically incorrect. In our uniaxial tensile tests on industrial pure aluminum at an ultralow strain rate of $\sim 10^{ - 7} /{\text{s}}$, we observed that SPD accompanied by grain refining significantly softened the material. The fundamental strength effective for real structures and structural materials should mean an eternal capability to bear stresses caused by external forces, which is independent of time, that is, athermal. We tried to extract quantitatively the athermal (time-independent) strength from the total strength measured in uniaxial tensile tests under the assumption that the total stress can be additively divided into athermal and thermal (time-dependent) components. As a result of systematic experimental investigation, we found that the athermal strength is significantly reduced by SPD and then markedly increased by subsequent low-temperature annealing. In addition, we confirmed that SPD promotes an increase in the time dependence (viscosity) of the material and that subsequent annealing removes most of the viscosity caused by SPD. The material processed by SPD acquires its prominent time-independent strength after low-temperature annealing.

## Introduction

It has been considered over the past two decades that severe plastic deformation (SPD) significantly improves the strength of metals^1–9^ and induces peculiar phenomena not observed in conventional metals, such as hardening by annealing^9^. Generally, SPD refines crystal grains to the micron or submicron scale. It has been believed that the main cause of strengthening by SPD is *grain refining effects*. This understanding originated from an expanded interpretation of the conventional Hall–Petch empirical relation^10,11^ (that is, the yield strength of metals linearly increases with the reciprocal of the square root of their grain size). On the other hand, the metals processed by SPD have a high dislocations density. Several researchers have insisted that the high strength of the SPD-processed metals is primarily governed by the dislocation density rather than the grain refining effects^12,13^. Thus, there are several different views regarding the causes of the high strength of the SPD-processed metals, and the mechanisms and physics of these SPD-processed metals are not yet understood completely.

Most of the studies carried out thus far for clarifying the strengthening mechanisms of the SPD-processed metals have not taken into account the time-dependent nature of the material behaviors^13–17^. The results of several studies^18–21^ have suggested that since SPD tends to promote an increase in viscosity as well as the stress relaxation characteristics of the materials, the time-dependent nature will be an important factor in understanding the physics correctly. In general, the fundamental *strength* effective for real structures and structural materials should mean an eternal capability to bear stresses caused by external forces. Such strength must be independent of time, that is, *athermal*.

In the present work, we attempted to extract quantitatively the athermal strength from the total strength measured in uniaxial tensile tests under the assumption that the total stress can be additively divided into athermal (time-independent) and thermal (time-dependent) components. An industrial pure aluminum (JIS A1070-H, 99.7% purity), one of the most popular and basic metals, was adopted as the target material. Equal-channel angular pressing (ECAP)^1,2^ was employed as the SPD processing method (Supplementary Fig. S1(a)–(b)). As a result of our systematic experimental study, we found that the athermal strength is significantly reduced by SPD and then markedly increased by subsequent low-temperature annealing. Additionally, we confirmed that SPD promotes a tremendous increase in the time-dependence (viscosity) of the material and that subsequent low-temperature annealing removes most of the viscosity caused by SPD.

## Results and Discussion

### Decrease in flow stress of SPDed samples at an ultralow strain rate

**Figure 1:**
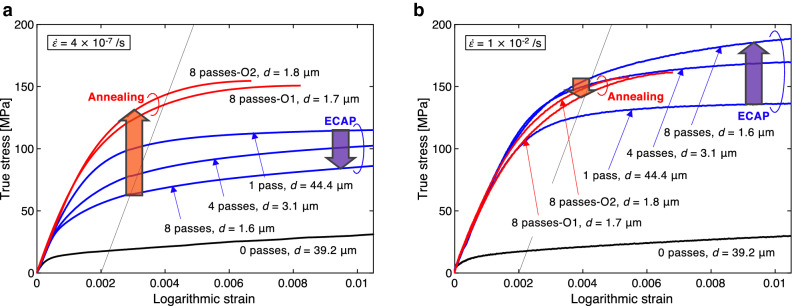
Experimental curves of true stress versus logarithmic strain in uniaxial tension at strain rates of (**a**) $\dot{\varepsilon } = 4 \times 10^{ - 7} /{\text{s}}$ and (**b**) $\dot{\varepsilon } = 1 \times 10^{ - 2} /{\text{s}}$. As-ECAP-processed samples and samples first processed with 8 ECAP passes and subsequently subjected to low temperature annealing were used. The average crystal grain sizes “*d”* shown in the graphs were taken from Ref. 13. Apparently, *softening by SPD* and *hardening by annealing* are observed for $\dot{\varepsilon } = 4 \times 10^{ - 7} /{\text{s}}$, while *hardening by SPD* and *softening by annealing* are observed for $\dot{\varepsilon } = 1 \times 10^{ - 2} /{\text{s}}$.

Figure 1 shows the results of tensile tests on samples processed with different numbers of ECAP passes (henceforth, the names of these samples are referred to as “0 passes”, “1 pass”, “4 passes” and “8 passes”) and samples first processed with 8 ECAP passes and subsequently annealed at 175 °C for 0.5 h or 6 h (henceforth referred to as “8 passes-O1” and “8 passes-O2”, respectively). The sample “0 passes” was the fully annealed one taken as the starting material. In the graphs, the grain sizes (*d*) measured^13^ by a standard electron backscatter diffraction (EBSD) method are also indicated. In ECAP processing, one ECAP pass introduces an equivalent strain (defined as the magnitude of uniaxial logarithmic strain) of approximately 1 into the sample. In the tensile tests, two tensile strain rates ($\dot{\varepsilon }$) were considered: one is an extraordinarily low strain rate of $4 \times 10^{ - 7} /{\text{s}}$, which may be close to the lowest limit that can be realized with a standard tensile testing machine, and the other is a strain rate of $1 \times 10^{ - 2} /{\text{s}}$, which lies within a range generally used for normal tensile tests. The imposed strain rates were controlled as strictly as possible by a feedback control method with wire strain gages or an extensometer (Supplementary Figs S1(c)–(e)). In the low-strain-rate tests (Fig. 1(a), Supplementary Fig. S2(a), $\dot{\varepsilon } = 4 \times 10^{ - 7} /{\text{s}}$), the tensile flow stress of the sample “0 passes” was quadrupled by the first ECAP processing. In the subsequent ECAP processes, the flow stress was markedly decreased. Significant *softening by SPD* was observed for 1 to 8 ECAP passes. We did not observe any *grain size strengthening* in the as-ECAP-processed samples. The subsequent low-temperature annealing approximately doubled the flow stress of the sample “8 passes” (see the curves for the samples “8 passes-O1” and “8 passes-O2” in Fig. 1(a)).

Figure 1(b) shows curves of true stress versus logarithmic strain for the normal strain rate tests (Supplementary Fig. S2(b), $\dot{\varepsilon } = 1 \times 10^{ - 2} /{\text{s}}$). Apparently, repeated ECAP processes gradually augment the strength of the sample, but the subsequent annealing slightly decreases it. Thus far, we have frequently observed stress–strain curves similar to those shown in Fig. 1(b) in the literature (e.g. Refs. 22–29), so it may have been concluded that grain refining promoted by SPD itself strengthens the material. However, it is obvious that this understanding is erroneous as now our Fig. 1(a) shows softening by SPD.

Huang et al.^9^ reported that slight hardening by low-temperature annealing occurred in industrial pure aluminum (99.2% purity) processed by accumulative roll bonding (ARB, one of the SPD processing methods). They only used a fixed strain rate of $\sim 4 \times 10^{ - 4} /{\text{s}}$. Thus, they would have observed an intermediate phenomenon between significant strengthening and slight softening by annealing, as seen in our Fig. 1(a,b).

### Decomposition of flow stress into athermal and thermal components

The observation that the flow stress varies depending on the imposed strain rate means that the material has time dependence (that is, viscosity). We assume that the observed flow stress $\sigma$ is the sum of an athermal (time-independent) component, $\sigma_{{\text{i}}}$, and a thermal (time-dependent) component, $\sigma^{*}$^30^, as

1 $$ \sigma = \sigma_{{\text{i}}} + \sigma^{*} . $$

**Figure 2:**
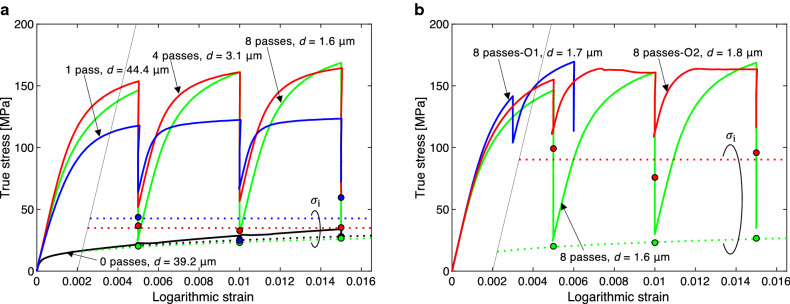
Experimental curves of true stress versus logarithmic strain in uniaxial tension obtained in stress relaxation tests: (**a**) fully annealed and as-ECAP-processed samples (0, 1, 4 and 8 passes); (**b**) samples first processed with 8 ECAP passes and subsequently subjected to low-temperature annealing and as-ECAP-processed sample (8 passes). As-ECAP-processed samples exhibit tremendous stress relaxation. Solid circles and dotted lines indicate the athermal stresses ($\sigma_{{\text{i}}}$) estimated utilizing Eq. (2). For simplicity, the dotted lines less than the 0.2% proof stress points were not indicated.

**Figure 3:**
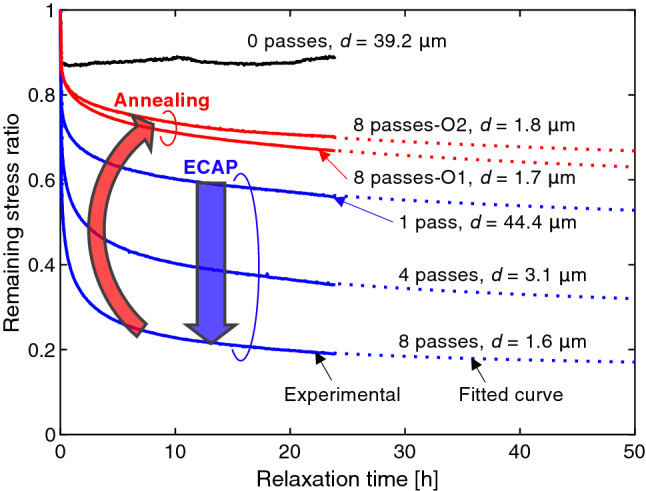
Typical stress relaxation behavior with respect to time (for relaxation tests at a logarithmic strain of 1%; only for the sample “8 passes-O1”, the result at a logarithmic strain of 0.6% is shown). The vertical axis indicates the ratio of the remaining stress during relaxation to the stress at the beginning of relaxation. Curves fit to Eq. (2) for the evaluation of athermal stress $\sigma_{{\text{i}}}$ are also shown.

To evaluate $\sigma_{{\text{i}}}$, stress relaxation tests were carried out. The asymptotic limit of $\sigma$ after a lapse of sufficiently long time in the stress relaxation test is assumed to be an approximation of $\sigma_{{\text{i}}}$. Figure 2 shows the results of the stress relaxation tests at three elongation stages (at logarithmic strains ($\varepsilon$) of 0.005, 0.01, and 0.015), except for the sample “8 passes-O1”, which encountered an early breakage. In these tests, we controlled the speed of the cross head of the testing machine to be constant during tensile loading. The actual logarithmic strain rates just before the beginning of relaxation, which determined the stress value at the beginning of relaxation, were in the range of $2 \times 10^{ - 4} /{\text{s}}$ to $1 \times 10^{ - 3} /{\text{s}}$ (Supplementary Fig. S2(c)). However, during the relaxation tests, the strain rate, i.e. the variation in strain, was imposed to be zero using a feedback control technique. In each relaxation test, the whole relaxation time was set to be 24 h. As seen in Fig. 2(a), the amount of stress relaxation increased with the number of ECAP passes. It is noteworthy that, after relaxation for 24 h, the stress of the sample “8 passes” dropped to a value nearly equal to the flow stress of the fully annealed sample “0 passes”. On the other hand, the degrees of stress relaxation of the annealed samples, “8 passes-O1” and “8 passes-O2”, were significantly smaller than that of the sample “8 passes”, as seen in Fig. 2(b). A typical relaxation behavior with respect to time is depicted in Fig. 3 (other results are shown in Supplementary Figs S3(a)–(b)). The vertical axis indicates the ratio of the remaining stress during the relaxation test to the stress at the beginning of relaxation. Except for the sample “0 passes”, the stresses continued to decrease even after 24 h. To evaluate $\sigma_{{\text{i}}}$, which is assumed to equal the limit of relaxation, the following approximation function is introduced:

2 $$\sigma = \sigma_{{\text{i}}} - \left( {\sigma_{{\text{i}}} - \sigma_{{\text{b}}} } \right){\exp}\left\{ { - \left( {kt} \right)^{c} } \right\}.$$

Here,* σ*_i_,* σ*_b_,* k* and* c* are fitting parameters and* t* is time. Dotted curves in Fig. 3 that seem to be extensions of the experimental curves are the results of fitting by Levenberg–Marquardt method (Supplementary Table S1(a)). The evaluated $\sigma_{{\text{i}}}$ values are indicated in Fig. 2 by solid circles. For the sample “0 passes”, the stress after relaxation for 24 h was assumed to be $\sigma_{{\text{i}}}$ since the decrease in flow stress stopped soon after starting the relaxation test. In principle, a curve that connects three $\sigma_{{\text{i}}}$ values for each specimen determines $\sigma_{{\text{i}}} \left( {\varepsilon_{{\text{p}}} } \right)$ as a function of the plastic strain $\varepsilon_{{\text{p}}}$. However, the three $\sigma_{{\text{i}}}$ values do not exhibit a monotonic variation, except for the samples “0 passes” and “8 passes”. The reason for the nonmonotonic variation in $\sigma_{{\text{i}}}$ is not understood at present. For the samples “0 passes” and “8 passes”, an *n*-th power law ($\sigma_{{\text{i}}} = F(\varepsilon_{{\text{p}}} )^{n} )$ is used to represent $\sigma_{{\text{i}}}$ as a function of $\varepsilon_{{\text{p}}}$, whereas for the other samples, the average of the three $\sigma_{{\text{i}}}$ values is taken to quantify an approximate $\sigma_{{\text{i}}}$, which is constant within the present small strain range (Supplementary Table S1(b)). The dotted lines shown in Fig. 2(a,b) are the evaluated athermal stresses, $\sigma_{{\text{i}}} (\varepsilon_{{\text{p}}} )$. The annealed sample “8 passes-O1” has only two data points for the stress relaxation tests, and thus, $\sigma_{{\text{i}}}$ was not quantified owing to doubt regarding reliability. It is expected that the $\sigma_{{\text{i}}}$ of the sample “8 passes-O1” will be close to that of the sample “8 passes-O2”. The above procedure for evaluating $\sigma_{{\text{i}}}$ was based on the premise that the material properties before and during stress relaxation are identical. We compared stresses observed in the low-strain-rate tests (Fig. 1(a)) and in the relaxation tests at the moments when the same plastic strain rate occurred. The relative difference between the stress values in the tensile tests and in the relaxation tests at the same plastic strain rate was within $\pm 20\%$ (mostly $\pm 10\%$; Supplementary Fig. S4). On the basis of this observation, the evaluated $\sigma_{{\text{i}}}$ values are considered to be fair approximations of the athermal strengths of the samples. Strictly, the internal microstructure and mechanical properties of the material would vary to some extent during the stress relaxation tests. Although this might affect the quantitative evaluation of $\sigma_{{\text{i}}}$, we believe that variation due to such unaccounted factors is not sufficiently significant as to overturn the major conclusion of the present study.

**Figure 4:**
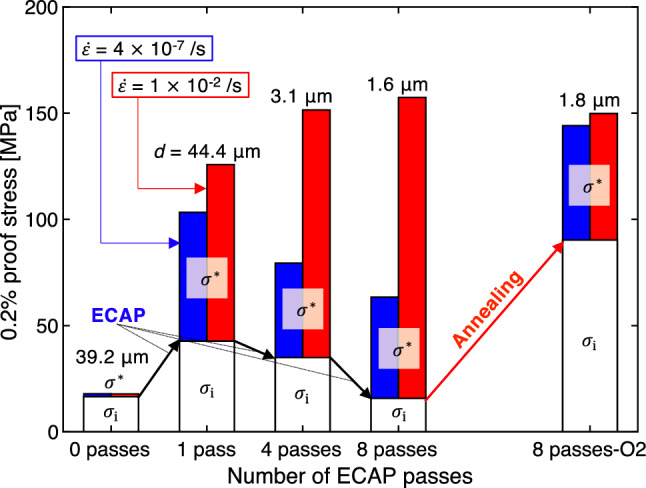
Breakdown of 0.2% proof stress into athermal component $\sigma_{{\text{i}}}$ and thermal component $\sigma^{*}$ for uniaxial tension tests at $\dot{\varepsilon } = 4 \times 10^{ - 7} /{\text{s}}$ and $\dot{\varepsilon } = 1 \times 10^{ - 2} /{\text{s}}$. SPD reduces athermal stress, while annealing augments it.

Figure 4 shows the measured flow stress $\sigma$ and its breakdown into $\sigma_{{\text{i}}}$ and $\sigma^{*}$ at a plastic strain of 0.2%. The thermal stresses ($\sigma^{*}$) were determined by subtracting the evaluated $\sigma_{{\text{i}}}$ from the flow stress $\sigma$ measured in the tensile tests at the low ($\dot{\varepsilon } = 4 \times 10^{ - 7} /{\text{s}}$; Fig. 1(a)) and normal ($\dot{\varepsilon } = 1 \times 10^{ - 2} /{\text{s}}$; Fig. 1(b)) strain rate. The values $\sigma$ (or $\sigma_{{\text{i}}}$) at a plastic strain of 0.2% (i.e., 0.2% proof stress or 0.2% athermal stress) were determined at the intersections between stress–strain curves in Fig. 1(a,b) (or dotted lines in Fig. 2 for $\sigma_{{\text{i}}}$) and narrow solid lines that indicate a supposed elastic material response with a Young’s modulus of 69 GPa. During the first ECAP processing, the amount of $\sigma_{{\text{i}}}$ was approximately doubled. The subsequent repeated ECAP processes significantly reduced $\sigma_{{\text{i}}}$. For the sample “8 passes”, $\sigma_{{\text{i}}}$ eventually became almost the same as that for the fully annealed material “0 passes”. In the meanwhile, the grain size decreased from ~ 40 μm to 1.6 μm with the repeated ECAP processes. The grain refining promoted by SPD never contributes to the increase in the athermal strength of the material. In Ref. 13, for a same-grade material, it was shown that the dislocation density reached its maximum after the first ECAP process, then it largely decreased with subsequent repeated ECAP passes. This suggests that the athermal strength is mainly governed by the dislocation density. The low-temperature annealing (175 °C for 6 h) augmented the athermal stress more than five times (the sample “8 passes-O2”). According to Ref. 13, the low-temperature annealing (175 °C for 6 h) results in a small decrease in dislocation density. Thus, it is considered that other strengthening mechanisms rapidly emerged during the low-temperature annealing process. The rearrangement of the dislocations and a change in the microstructure of the grain boundaries may be candidate mechanisms. This is uncertain at present and should be subjected to further investigations.

With increasing number of ECAP passes, the thermal stress component $\sigma^{*}$ in the low-strain-rate tests ($\dot{\varepsilon } = 4 \times 10^{ - 7} /{\text{s}}$) decreased, whereas $\sigma^{*}$ in the normal-strain-rate tests ($\dot{\varepsilon } = 1 \times 10^{ - 2} /{\text{s}}$) increased. The difference between the amounts of the thermal stress component in the low- and normal-strain-rate tests increased with the number of ECAP passes. This means that the viscosity (time dependence) of the material is markedly enhanced by SPD. After the low-temperature annealing, the difference between the amounts of the thermal stress component in the low- and normal-strain-rate tests significantly decreased. The time dependence was suppressed by annealing.

## Conclusions

In the present experimental study on industrial pure aluminum, it is emphasized that SPD itself does not strengthen the material. There is evidence that SPD led to significant softening at an ultralow strain rate of $\sim 10^{ - 7} /{\text{s}}$ (Supplementary Fig. S5 shows the reproducibility). This is caused by the fact that the athermal strength, which is independent of time and is the eternal stress bearing capability, decreases with successive SPD processes. The athermal strength significantly increases after low-temperature annealing. It is also clarified that SPD greatly augments the material viscosity, while low-temperature annealing significantly suppresses it. Thus far, it has generally been believed that ultrafine crystal grains produced by SPD contribute directly to the fundamental strengthening of materials. The results of the present study show that this understanding is incorrect. SPD provides the material (aluminum in this study) with grain refining, decreases the athermal strength, and increases the viscosity. The material processed by SPD acquires its prominent time-independent strength after low-temperature annealing.

## Methods

### Material

An industrial pure aluminum (JIS A1070-H, 99.7% purity; a commercial product) was used.

### Sample preparation by severe plastic deformation and heat treatment

We used equal-channel angular pressing (ECAP) as the SPD processing method^1,2^ using a die with a circular channel with a diameter of 10 mm, an inner angle of bend, $\phi$, of 90° and an outer angle of bend, $\psi$, of 36.87° (Supplementary Fig. S1(a)). Molybdenum disulfide paste was used as a lubricant. The ECAP process called the ‘route Bc’^31^ was adopted, which is accompanied by the rotation of the sample by 90˚ about the sample axis at each ECAP pass. The route Bc procedure is known to give approximately equiaxial crystal grains^31^.

The equivalent strain introduced into the sample is approximately expressed^2^ by

3 $$ \varepsilon^{{{\text{ECAP}}}} = \frac{n}{\sqrt 3 }\left\{ {2{\cot}\left( {\frac{\phi }{2} + \frac{\psi }{2}} \right) + \psi {\text{cosec}}\left( {\frac{\phi }{2} + \frac{\psi }{2}} \right)} \right\}, $$

where *n* is the number of ECAP passes.

Before the ECAP operations, all A1070-H rods (with a diameter of 9.95 mm and a length of 60 mm or 120 mm) were annealed at 425 °C for 1 h using an electric furnace (Isuzu EPDS-2 K) in an air atmosphere. Each annealed rod was the starting material for the subsequent ECAP operations, which was referred to as the “0 passes” sample. The ECAP operation was carried out manually at room temperature. For one ECAP pass, it takes 120 to 180 s, and thus the average crosshead speed of the testing machine (Tokyo Koki hydraulic universal testing machine) was 0.3 to 0.5 mm/s approximately.

To investigate the effects of heat treatment on the mechanical properties, some of the as-ECAP-processed samples “8 passes” were subjected to annealing at 175 °C for 0.5 h and at 175 °C for 6 h using an electric furnace (Advantec DRV220DA). They were “8 passes-O1” and “8 passes-O2”, respectively.

### Low- and normal-strain-rate tensile tests ($\dot{\user2{\varepsilon }} = 4 \times 10^{ - 7} \user2{ }/{\mathbf{s}}$ and $\dot{\user2{\varepsilon }} = 1 \times 10^{ - 2} \user2{ }/{\mathbf{s}}$)

For tensile tests, the ECAP-processed samples (rod-shaped) were machined into dumbbell-shaped specimens with threaded grips. The dimensions of the specimens for the low-strain-rate tests ($\dot{\varepsilon } = 4 \times 10^{ - 7} /{\text{s}}$) and for the normal-strain-rate tests ($\dot{\varepsilon } = 1 \times 10^{ - 2} /{\text{s}}$) were illustrated in Supplementary Fig S1(d) and (e), respectively. The tensile tests were carried out at room temperature using a tensile testing machine (Shimadzu AG–X plus 300kN) in which an in-house made chucking device with thread-cut parts was installed to hold the specimen.

In the low-strain-rate tests ($\dot{\varepsilon } = 4 \times 10^{ - 7} /{\text{s}}$), the strain rate was controlled by a feedback control method with a contact extensometer (Shimadzu SSG50-10H) for the first 7,200 s. After that, the strain rate was controlled by a constant crosshead speed of 0.00137 mm/min. The actual imposed strain rate on the specimens against logarithmic strain is shown in Supplementary Fig. S2(a).

In the normal-strain-rate tests ($\dot{\varepsilon } = 1 \times 10^{ - 2} /{\text{s}}$), the strain rate was controlled throughout the test by a feedback control method utilizing an opposite arm half bridge with two 3-wire active gauges (TML FLA-2–23) glued on the specimen surface. The actual imposed strain rate on the specimens against logarithmic strain is shown in Supplementary Fig. S2(b).

### Stress relaxation tests

The stress relaxation tests at three elongation stages (at logarithmic strains ($\varepsilon$) of 0.005, 0.01, and 0.015) were carried out. Toward each elongation stage, tensile loading was governed by a constant crosshead speed of 0.48 mm/min. Thus, the actual strain rates of the specimens varied during tensile loading as shown in Supplementary Fig. S2(c). The actual strain rates just before the beginning of relaxation, which determined the stress value at the beginning of relaxation, were in the range of $2 \times 10^{ - 4} /{\text{s}}$ to $1 \times 10^{ - 3} /{\text{s}}$. During the relaxation tests, the variation in strain was kept to be zero using the feedback control with the opposite arm half bridge with two 3-wire active gauges (TML FLA-2–23). Supplementary Table S1(a) shows curve-fitting details of the experimental stress relaxation behaviors using Eq. (2). Supplementary Table S1(b) shows curve-fitting details of the athermal stress–logarithmic plastic strain relations using *n*-th power law.

### Identification of athermal stress

Our method for identifying $\sigma_{{\text{i}}}$ using the stress relaxation tests was based on the assumption that the material properties before and during stress relaxation were nearly identical. To examine the validity of this assumption, we compared stresses observed in the low-strain-rate tests and in the relaxation tests at the moments when the same plastic strain rate occurred. We extracted the true stresses and strain rates at strains of 0.005, 0.01 and 0.015 from the results of the low-strain-rate tests with $\dot{\varepsilon } = 4 \times 10^{ - 7} /{\text{s}}$ (Fig. 1(a)). On the other hand, from the curves fitted to the stress relaxation test results (Fig. 3 and Supplementary Fig. S3(a) and (b), we computed relationships between the true stress and the plastic strain rate during stress relaxation. The relative difference (*RD*, defined by Eq. (4)) between the stress values in the tensile tests and in the relaxation tests at the same plastic strain rate was within $\pm 20\%$ (mostly $\pm 10\%$; Supplementary Fig. S4). The definition of the relative difference, *RD*, is

4 $$ RD = \frac{{\sigma_{{{\text{relax}}}} - \sigma_{{{\text{tens}}}} }}{{ \sigma_{{{\text{tens}}}} }} , $$

where $\sigma_{{{\text{tens}}}}$ and $\sigma_{{{\text{relax}}}}$ are the true stresses observed in the low-strain-rate tensile tests and in the relaxation tests, respectively, at the moments when the same plastic strain rate value was recorded.

## Supplementary information

Supplementary file1 (PDF 521 kb)

## Data Availability

The data that support the findings of this study are available from the corresponding authors on reasonable request.
