# Supplementary material for: Athermal strength of pure aluminum is significantly decreased by severe plastic deformation and it is markedly augmented by subsequent annealing
Source: Sci Rep. 2020 Aug 24;10:14090. doi: 10.1038/s41598-020-70160-5 (PMC7445292; doi:10.1038/s41598-020-70160-5)
Supplement: Supplementary file 1 — Supplementary file1 (PDF 521 kb) [file 41598_2020_70160_MOESM1_ESM.pdf]

Supplementary information

Athermal strength of pure aluminum is significantly decreased by severe plastic deformation and it is markedly augmented by subsequent annealing

Takayuki Koizumi[1*], Anna Kurumatani[2] and Mitsutoshi Kuroda[2*]

[1] *Faculty of Human Resources Development, Polytechnic University of Japan, Tokyo 187-0035, Japan*

[2] *Graduate School of Science and Engineering, Mechanical Engineering, Yamagata University, Yamagata 992-8510, Japan*

* Corresponding authors: t-koizumi@uitec.ac.jp and kuroda@yz.yamagata-u.ac.jp

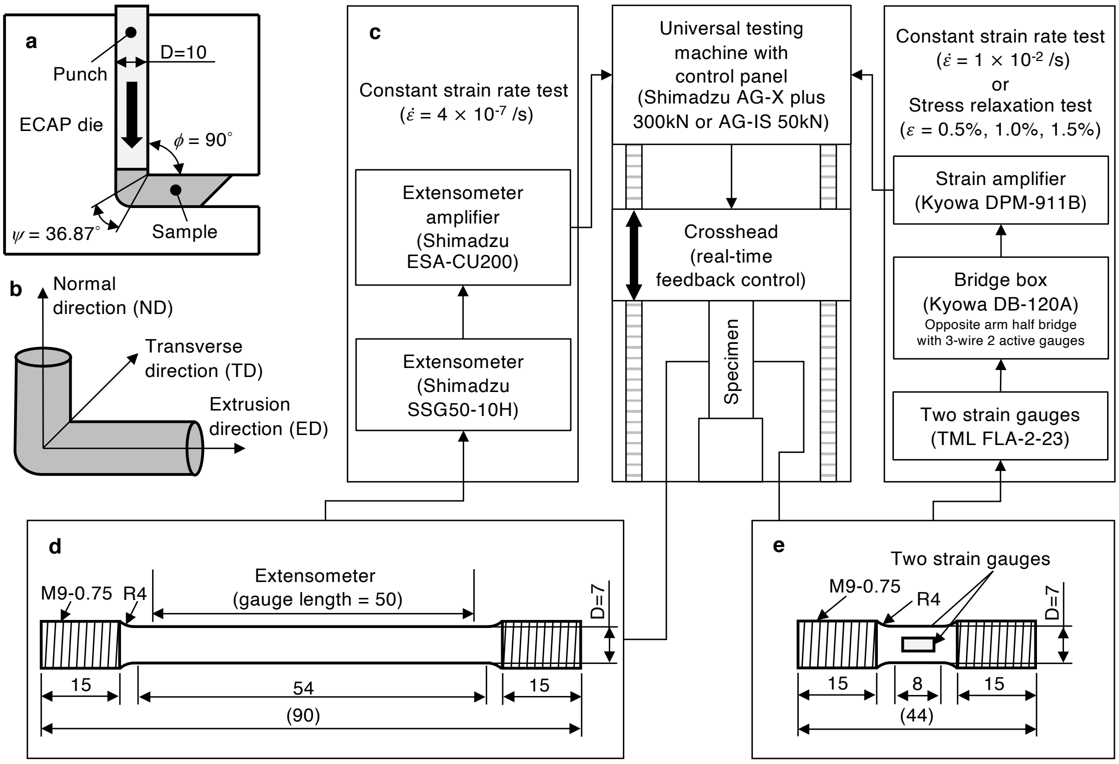

Supplementary Figure S1. Schematic diagrams for experimental procedure. (a) Die used for ECAP processing; (b) Sample coordinates for ECAP processing; (c) Methods for controlling the strain rates during the tensile and stress relaxation tests; (d) Shape and dimensions of the specimen for the low-strain-rate tensile tests; (e) Shape and dimensions of the specimen for the normal-strain-rate tensile and stress relaxation tests.

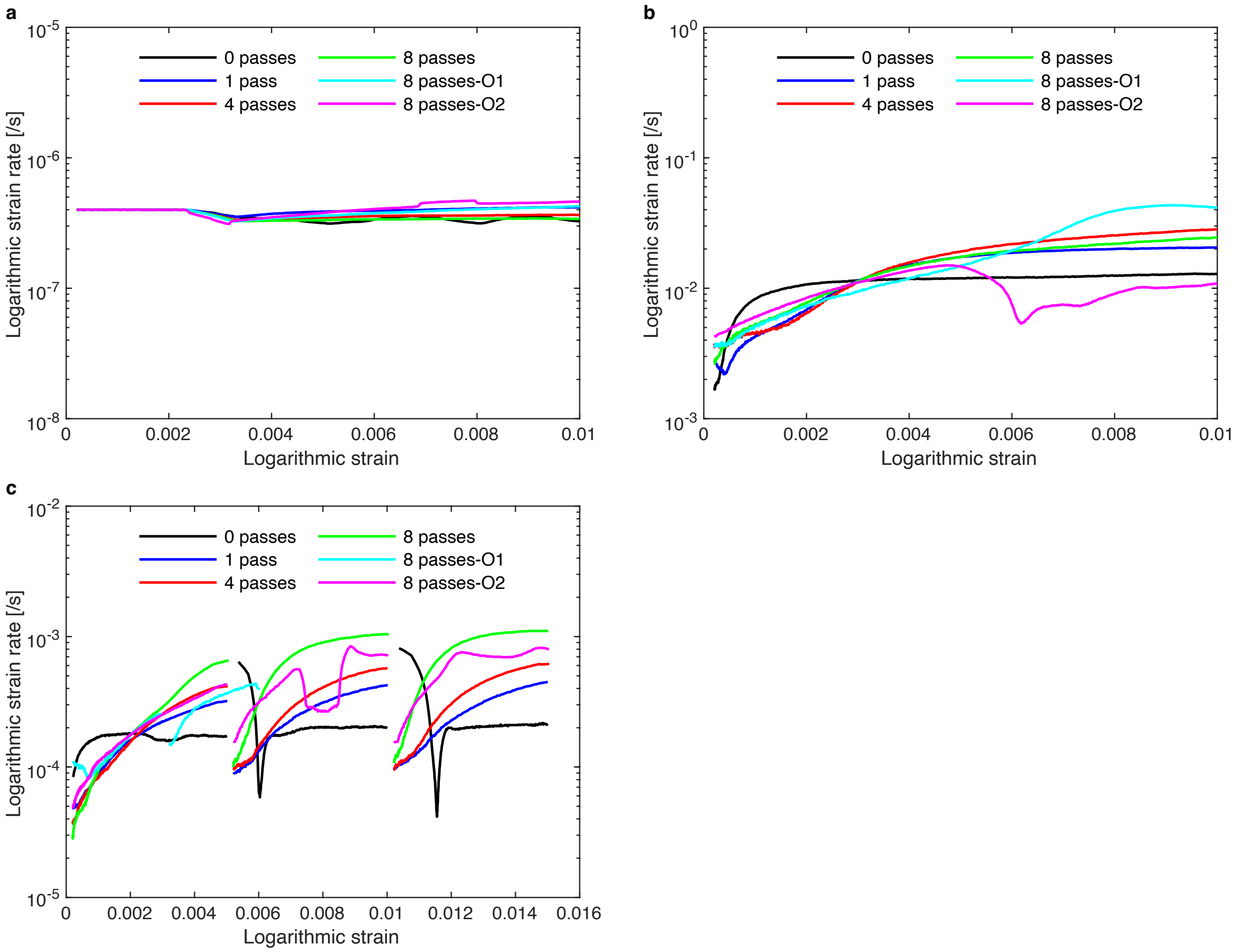

Supplementary Figure S2. (a) Actual strain rates for the command value of $\dot{\varepsilon} = 4 \times 10^{-7}$ /s in the feedback control method; (b) Actual strain rates for the command value of $\dot{\varepsilon} = 1 \times 10^{-2}$ /s in the feedback control method; (c) Actual strain rates during tensile loading toward the stages where the stress relaxation tests started (these loading speeds were governed by a constant crosshead speed of the testing machine). In these graphs, part of dense raw experimental data has been thinned out and the curves are smoothed out using a central moving average in order to remove effects of unavoidable noise.

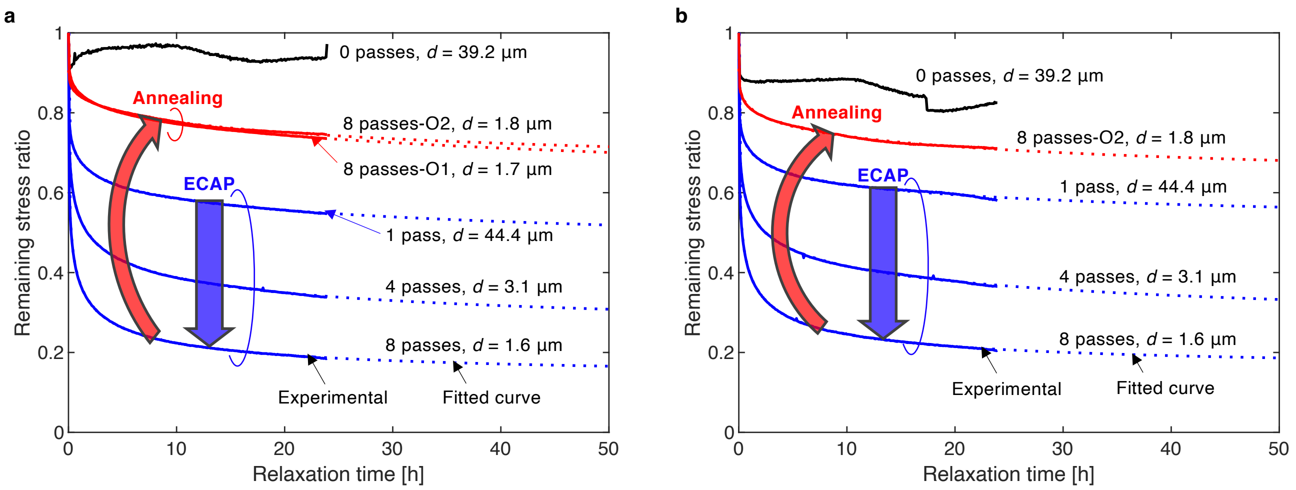

Supplementary Figure S3. (a) Stress relaxation behavior with respect to time for the relaxation tests at a logarithmic strain of 0.5% (only for the sample “8 passes-O1”, the result at a logarithmic strain of 0.3% is shown); (b) Stress relaxation behavior with respect to time for the relaxation tests at a logarithmic strain of 1.5 %. The both results are similar to those shown in Fig. 3 in the main text.

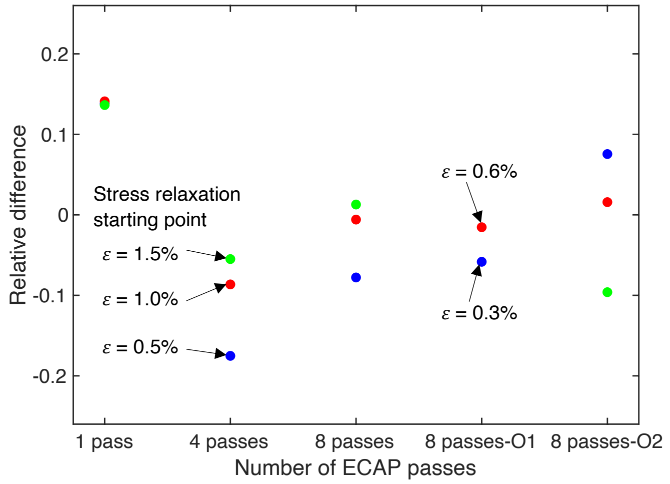

Supplementary Figure S4. Relative difference between stress values in the tensile tests and in the relaxation tests when the same plastic strain rate occurred.

Supplementary Table S1. (a) Details of curve-fitting of the experimental stress relaxation behaviors using Eq. (2); (b) Details of curve-fitting of the athermal stress–logarithmic plastic strain relation using *n*-th power law.

a Curve fit parameters of stress relaxation tests at three elongation stages; σ [MPa] $= \sigma_i - (\sigma_i - \sigma_b)\exp\{-(kt)^c\}$, t: relaxation time [s]

Number of ECAP passes	ε = 0.5% (0.3%; 8 passes-O1)				ε = 1.0% (0.6%; 8 passes-O1)				ε = 1.5%				Coefficient of determination R^2		
	σ_i [MPa]	σ_b [MPa]	c [−]	k [s^{-1}]	σ_i [MPa]	σ_b [MPa]	c [−]	k [s^{-1}]	σ_i [MPa]	σ_b [MPa]	c [−]	k [s^{-1}]	ε = 0.5%	ε = 1.0%	ε = 1.5%
0 passes	σ_i = 20.96 (remaining stress at 24 h)				σ_i = 25.65 (remaining stress at 24 h)				σ_i = 28.08 (remaining stress at 24 h)				–		
1 pass	43.68	118.24	1.83E-01	4.25E-05	24.83	119.01	1.69E-01	2.28E-06	59.58	115.42	2.27E-01	5.97E-05	0.9996	0.9993	0.9965
4 passes	36.74	168.32	2.24E-01	3.39E-04	32.75	185.48	1.85E-01	3.05E-04	35.32	184.31	1.89E-01	2.41E-04	0.9997	0.9995	0.9994
8 passes	20.14	176.20	2.29E-01	1.57E-03	23.05	187.29	2.33E-01	1.39E-03	26.58	193.15	2.35E-01	1.19E-03	0.9998	0.9998	0.9998
8 passes-O1	12.94	141.70	2.03E-01	6.09E-08	0	166.82	1.96E-04	9.04E-08	–				0.9999	0.9999	–
8 passes-O2	99.25	140.56	4.01E-01	9.97E-06	75.85	149.15	2.60E-01	2.90E-06	95.88	148.71	3.20E-01	1.08E-05	0.9975	0.9981	0.9972

b

Number of ECAP passes	Curve fit parameters of *n*-th power law for athermal stresses; σ_i [MPa] $= F(\varepsilon_p)^n$			σ_i [MPa] (ε_p = 0.2%)
	F [MPa]	n [−]	Coefficient of determination R^2	
0 passes	86.37	0.266	0.9951	16.52
1 pass	42.70	0	–	42.70
4 passes	34.94	0	–	34.94
8 passes	75.76	0.253	0.9722	15.76
8 passes-O1	–	–	–	–
8 passes-O2	90.33	0	–	90.33

Reproducibility of the main result.

To confirm reproducibility of the phenomenon "softening by SPD", we performed independently another series of the low-strain-rate tensile tests with $\dot{\varepsilon} = 1 \times 10^{-6}$ /s using the other testing machine we have (Shimadzu AG-IS 50kN). The dimensions of the specimen were similar to those shown in Fig. S1(e), but with an extended parallel region length of 80 mm. The results are shown in Fig. S5, which are very similar to the results for $\dot{\varepsilon} = 4 \times 10^{-7}$ /s (Fig. 1(a) in the main text).

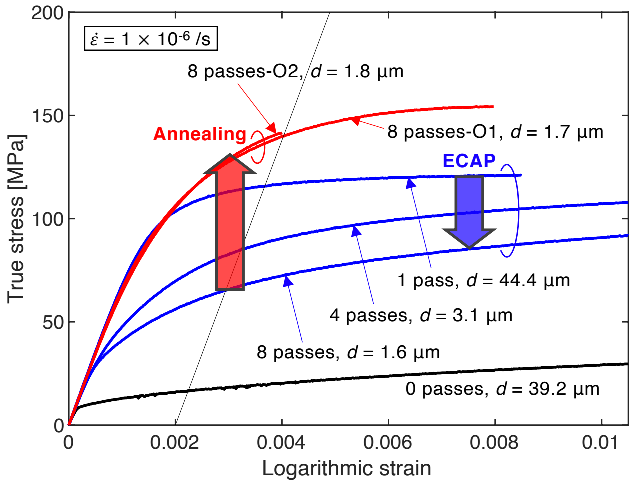

Supplementary Figure S5. Results for an additional set of low-strain-rate tensile tests with $\dot{\varepsilon} = 1 \times 10^{-6}$ /s. The purpose of these additional experiments was confirmation of the reproducibility of the phenomenon "softening by SPD" exhibited in Fig. 1(a) in the main text.
